# Copeptin under glucagon stimulation

**DOI:** 10.1007/s12020-015-0783-7

**Published:** 2015-11-17

**Authors:** Krzysztof C. Lewandowski, Andrzej Lewiński, Elżbieta Skowrońska-Jóźwiak, Magdalena Stasiak, Wojciech Horzelski, Georg Brabant

**Affiliations:** Department of Endocrinology and Metabolic Diseases, Medical University of Lodz, Lodz, Poland; Department of Endocrinology and Metabolic Diseases, Polish Mother’s Memorial Hospital - Research Institute, Lodz, Poland; Faculty of Mathematics and Computer Science, University of Lodz, Lodz, Poland; Experimental and Clinical Endocrinology Med Clinic I, University of Luebeck, Ratzeburger Allee 160, 23538 Lübeck, Germany; Department of Endocrinology, The Christie Manchester Academic Health Science Centre, Wilmslow Rd, Manchester, M20 4BX UK

**Keywords:** Copeptin, Glucagon stimulation test, Hypopituitarism, GH, ACTH, Cortisol

## Abstract

Stimulation of growth hormone (GH) and adrenocorticotropic hormone (ACTH) secretion by glucagon is a standard procedure to assess pituitary dysfunction but the pathomechanism of glucagon action remains unclear. As arginine vasopressin (AVP) may act on the release of both, GH and ACTH, we tested here the role of AVP in GST by measuring a stable precursor fragment, copeptin, which is stoichiometrically secreted with AVP in a 1:1 ratio. ACTH, cortisol, GH, and copeptin were measured at 0, 60, 90, 120, 150, and 180 min during GST in 79 subjects: healthy controls (Group 1, *n* = 32), subjects with pituitary disease, but with adequate cortisol and GH responses during GST (Group 2, *n* = 29), and those with overt hypopituitarism (Group 3, *n* = 18). Copeptin concentrations significantly increased over baseline 150 and 180 min following glucagon stimulation in controls and patients with intact pituitary function but not in hypopituitarism. Copeptin concentrations were stimulated over time and the maximal increment correlated with ACTH, while correlations between copeptin and GH were weaker. Interestingly, copeptin as well as GH secretion was significantly attenuated when comparing subjects within the highest to those in the lowest BMI quartile (*p* < 0.05). Copeptin is significantly released following glucagon stimulation. As this release is BMI-dependent, the time-dependent relation between copeptin and GH may be obscured, whereas the close relation to ACTH suggests that AVP/copeptin release might be linked to the activation of the adrenal axis.

**Electronic supplementary material:**

The online version of this article (doi:10.1007/s12020-015-0783-7) contains supplementary material, which is available to authorized users.

## Introduction

Copeptin is a 39-amino-acids containing glycosylated peptide, derived from the C-terminal part of the AVP precursor [1]. In the process of proteolysis, the AVP precursor is processed to AVP, neurophysin II, and copeptin in equimolar amounts. Plasma concentrations of arginine vasopressin are technically difficult to determine due to the small molecular size and its binding to platelets. In contrast to AVP, copeptin remains stable for several days at room temperature in serum or plasma [2]. Copeptin serves as a *bona fide* biomarker of AVP release based on large studies and is very useful in the diagnosis of diabetes insipidus [3]. In particular, copeptin concentrations 45 min after injection of insulin during an insulin tolerance test (ITT) provide the best sensitivity and specificity for detection of diabetes insipidus [4].

AVP is involved not only in hemodynamics and osmoregulation, but also in the endocrine stress response. Corticotrophin-releasing hormone (CRH) and AVP appear to have a synergistic effect resulting in ACTH and cortisol release [5, 6]. That is why measurements of copeptin have been proposed as a prognostic marker in an emergency situation, particularly in patients with acute diseases such as lower respiratory tract infection, heart disease, and stroke [7, 8]. Moreover, copeptin measurements seem to offer many advantages, being not very demanding or time-consuming, while at the same time being reliable and reproducible [8]. Hence, there are studies, where the authors suggested that stimulatory effects of AVP (as assessed by copeptin measurements) might be useful not only in testing for diabetes insipidus [4]. In addition, it may also be useful in the assessment of anterior pituitary function as shown by a significant copeptin stimulation following insulin induced hypoglycemia in a standard ITT [17]. Alternatively GST is used as a safe, reliable, and reproducible replacement to ITT to assess GH and ACTH/cortisol secretion. Currently, the underlying pathomechanism for the glucagon-dependent stimulation of anterior pituitary hormone release is unknown [9, 10]. As glucagon is rapidly increased following insulin-dependent hypoglycemia, we tested in the current study the hypothesis that a glucagon challenge may stimulate copeptin release and explain at least in part the ACTH***/***cortisol and GH response in GST.

## Patients and methods

The study involved 79 subjects (16 males), who had a GST performed in order to assess ACTH, cortisol, GH and copeptin secretion. The test involved measurements of ACTH, cortisol, GH, copeptin, and glucose before and after intramuscular administration of glucagon. The study group was further subdivided into healthy controls (Group 1, *n* = 32) and patients with various forms of pituitary disease. According to their results in the GST, we further subdivided the patient group into subjects with satisfactory cortisol and GH responses (Group 2, *n* = 29), and those who failed GST with regard either to GH, or cortisol secretion, or to both (Group 3, *n* = 18). Finally, from patients in Group 3, we selected subjects with diabetes insipidus (Group 3_A, *n* = 8), i.e., individuals displaying both anterior and posterior pituitary dysfunctions. Diagnoses of patients in groups 2 and 3 (*n* = 47 in total) included predominantly non-secreting pituitary macroadenomas after pituitary surgery. In Group 2, two patients suffered of an ACTH-secreting pituitary adenoma (cured), 2 of macroprolactinomas (controlled on dopamine agonists), 1 of traumatic brain injury (without diabetes insipidus), 1 of lymphocytic hypophysitis, 5 of acromegaly (1 still active, postsurgery), 1 of isolated childhood-onset GH deficiency, and 1 of isolated central diabetes insipidus, while the remaining (*n* = 16) had non-secreting pituitary adenomas. Clinical diagnoses in patients from Group 3 included craniopharyngioma (*n* = 3), dysgerminoma (*n* = 2), macroprolactinoma (*n* = 2), ACTH-secreting pituitary adenoma (cured, *n* = 1), acromegaly (cured, *n* = 1), Prader-Willy syndrome (*n* = 1), steroid-induced adrenal suppression (*n* = 1), and non-functioning pituitary adenomas (*n* = 7). Patients with hypopituitarism (all from Group 3) received hormone replacement therapy depending on the magnitude of the defect. This included hydrocortisone (*n* = 14), l-thyroxine (*n* = 12), intramuscular testosterone (*n* = 2), or oestrogens ± progestin (*n* = 3). As treatment with GH is not covered by the Polish state insurance, none of the patients where GH substituted. The average time after pituitary surgery ranged between 4 months and 10 years. There were no significant age differences between groups apart from a slightly increased BMI in patients with a history of pituitary disease (age: Group 1: 34.6 ± 14.4 years, Group 2: 40.7 ± 11.7 years, Group 3: 43.5 ± 13.6 years, *p* = 0.141; BMI: Group 1: 23.3 ± 4.7 kg/m^2^, Group 2: 25.9 ± 4.5 kg/m^2^, Group 3: 26.9 ± 7.0 kg/m^2^, *p* = 0.041, difference significant between Group 1 and 3 only).

### Glucagon test protocol

All our patients were normotensive and subjected an identical external stress, i.e., glucagon stimulation test was performed in the hospital setting in the morning on the second day of admission. All had normal renal function. GST was started at 8 am in a fasting state. Glucagon was administered intramuscularly at a dose of 1.0 mg (and 1.5 mg for those over 90 kg). Blood samples were taken before i.m. glucagon (0 min), and subsequently at 60, 90, 120, 150, and 180 min. Exclusion criteria to GST included diabetes mellitus and hyponatremia (plasma Sodium below 136 mmol/l). The cutoff value for a successful GST was defined as >3 ng GH/ml and >450 nmol cortisol/l (16.25 µg/dl) [10, 11].

### Assays

Measurements of cortisol and other hormones including free T4, free T3, TSH, LH, FSH, prolactin, testosterone, and oestradiol were performed by immunoassays on Roche Diagnostics COBAS e601 platform, while GH and ACTH were measured by immunoassays on Siemens IMMULITE 2000 XPi platform. Copeptin was measured with a new sandwich immunoassay, as described before [2, 12]. This assay has a lower detection limit of 0.4 pmol/l; functional assay sensitivity at <20 % interassay CV, <1 pmol/l [2]. All samples were assayed as a batch analyzed in one run by courtesy of ThermoFisher Scientific, Hennigdorf, Germany.

The study was approved by the Ethics Committee of the Polish Mothers’ Memorial Research Institute, Lodz, Poland.

### Statistical analysis

Statistical analysis was performed by means of MedCalc Software 12.6.1 software. Analysis of measured covariates was performed both by serial measurements method (area under the curve (AUC) calculation) and by ANOVA at distinct time points following glucagon stimulation. For nonparametric data, Kruskal–Wallis test was used instead. Correlation analyses were performed using Pearson coefficient or Spearman’s rank correlation. Wilcoxon test for paired samples was used for comparison of the parameters’ values for different times of measurement.

*p* values 0.05 were considered to indicate statistical significance.

## Results

### Overall copeptin response during GST

**Fig. 1:**
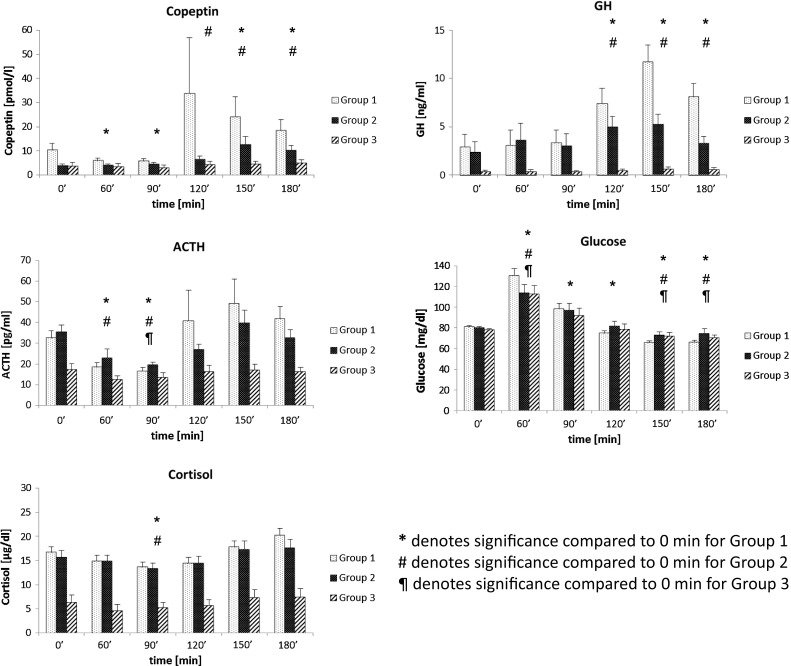
ACTH, Copeptin, and Growth Hormone concentrations during a glucagon stimulation test (GST). Patients were subdivided into Group 1 (healthy controls, *n* = 32), Group 2 (history of pituitary disease, but passed GST, *n* = 29), and Group 3 (unsatisfactory response during GST for GH, cortisol, or both, *n* = 18). *Vertical* lines denote standard errors of the means (SEMs)

Results of assessment of baseline hormonal parameters (thyroid function tests, gonadotropins, prolactin, and sex steroids) are presented in supplementary Table A. Basal copeptin concentrations were significantly higher in Group 1 than in Group 2 (*p* = 0.02) and in Group 3 representing patients who failed GST (Fig. 1; Table 1). In subjects who passed GST (Group 1 and Group 2), glucagon leads to a robust increase in copeptin concentrations 150 and 180 min following GST but this increase was attenuated in patients of Group 3 and failed when DI was present (Fig. 1; supplementary Table B).

**Table 1:** Copeptin, ACTH, cortisol, and growth hormone (GH) calculated by serial measurements method (area under the curve (AUC) calculation), i.e., a general linear model that calculates an integral for measured variables

	Group	Mean (area under the curve—arbitrary units)	95 % CI of the mean	SD	*p* value for all 3 group compared	*p* Group 1 versus Group 2	*p* Group 1 versus Group 3	*p* Group 2 versus Group 3
Copeptin	1	2627.3	1238.5–4016.1	3651.1	0.0034	0.3238	0.0008	0.0109
Copeptin	2	1620.4	896.3–2344.4	1674.4				
Copeptin	3	695.2	217.7–1172.7	896.1				
ACTH	1	4589.9	3754.9–5424.8	2110.7	<0.001	0.2593	0.0004	<0.0001
ACTH	2	4949.3	4278.7–5619.8	1550.7				
ACTH	3	2596.1	1558.077–3634.2	1874.5				
Cortisol	1	2802.8	2451.0–3154.6	942.1	<0.001	0.8892	<0.0001	<0.0001
Cortisol	2	2779.7	2358.838–3200.6	996.7				
Cortisol	3	1095.0	580.1–1609.9	1035.4				
GH	1	1054.7	529.117–1580.2	1328.6	<0.001	0.0041	<0.0001	0.0010
GH	2	496.6	260.986–692.1	498.5				
GH	3	75.2	19.9–130.5	99.8				

Group 1: controls—no pituitary disease, (*n* = 32), Group 2—pituitary disease, but passed glucagon stimulation test—GST (*n* = 29) and Group 3—pituitary disease failed GST—inadequate response of GH, cortisol or both (*n* = 18)

### Copeptin relationship to cortisol, ACTH, and GH

We next analyzed whether this copeptin increase was linked to ACTH or GH. In Group 1, copeptin significantly correlated with ACTH at the time of maximal copeptin release (120, 150, 180 min), but this correlation was only significant 150 min following glucagon stimulation in Group 2. Copeptin did not correlate with any other measured parameters (cortisol, GH or glucose; Table 2). Similarly, the maximum increment (i.e., ∆) for copeptin and ACTH in all subjects correlated significantly with the maximal increases of ACTH (ACTH ∆) but also with maximal increases of cortisol (cortisol ∆) (supplementary Table). In Group 2 and 3, no significant correlation was found, and in none of these analyses, GH was significantly altered. Interestingly, when all patients with normal posterior pituitary function were combined (*n* = 70), the maximum copeptin response at 150 min significantly correlated not only to ACTH (*r* = 0.317, *p* = 0.016) and cortisol (*r* = 0.405, *p* = 0.0012) but also to GH (*r* = 0.36, *p* = 0.006) and remained significant in a multiple regression model (*r* = 0.469 ± 0.0457, *p* < 0.0001 for ACTH_150 min_; *r* = 0.06992 ± 0.2868, *p* < 0.0185 for GH_150 min_).

**Table 2:** Correlations of Copeptin with ACTH, cortisol, and GH in Group 1 (healthy controls—no pituitary disease, *n* = 32), Group 2 (pituitary disease, but passed glucagon stimulation test—GST, *n* = 29), and Group 3 (pituitary disease failed GST—inadequate response of GH, cortisol or both, *n* = 18), statistically significant values (Pearson’s correlation coefficient is marked with asterisk (*) for *p* < 0.05)

Time	ACTH			Cortisol			GH			Glucose		
	Group 1	Group 2	Group 3	Group 1	Group 1	Group 3	Group 1	Group 2	Group 3	Group 1	Group 2	Group 3
0′	**0.695**	**0.494**	−0.118	−0.118	−0.118	−0.059	−0.118	0.143	−0.104	0.341	−0.066	0.389
60′	−0.065	0.253	−0.016	−0.016	−0.016	0.145	−0.016	0.340	−0.181	0.419	0.004	0.215
90′	−0.025	0.095	−0.143	−0.143	−0.143	0.0749	−0.143	0.146	−0.067	0.367	−0.137	0.263
120′	**0.929**	0.379	−0.072	−0.072	−0.072	−0.044	−0.072	−0.018	0.371	0.154	−0.473	0.103
150′	**0.866**	**0.614**	0.139	0.139	0.139	0.358	0.139	0.312	0.479	0.190	−0.309	0.271
180′	**0.399**	0.402	−0.087	−0.086	−0.086	0.406	−0.086	0.347	0.259	0.277	0.048	0.427

Statistical significance values are highlighted in bold for better visibility

### Copeptin secretion during GST and body mass index

As GH is well known to closely depend on body weight, we reanalyzed our data according to BMI and stratified our group in quartiles comparing the highest to the lowest BMI quartile. For this analysis, we combined all subjects who passed the GST in order to increase the power of the analysis. When comparing subjects with the lowest BMI quartile (i.e., BMI <20.82 kg/m^2^) to those with the highest BMI quartile (BMI >27.57 kg/m^2^), there was no BMI-dependent difference in ACTH and cortisol release. In contrast, the release of copeptin (and of GH) after was significantly lower in subjects within the highest BMI quartile (comparing baseline to 120 and 150 min of GST) (Table 3). Expectedly we observed significantly higher glucose levels in subjects with higher BMI at 90, 120, and 150 min of GST (Table 3). In contrast, when analyzed according to gender, no difference between male and female subjects was detected.

**Table 3:** Analysis of copeptin, growth hormone (GH), ACTH, cortisol, and glucose secretion in relation to body mass index (BMI) during glucagon stimulation test (Group 1 & 2, i.e., all subjects who passed glucagon stimulation test, *n* = 61)—comparison of the subjects with the lowest BMI quartile (BMI <20.82 kg/m^2^) versus those with the highest quartile (BMI >27.57 kg/m^2^)

Parameter	Time	BMI <20.82 kg/m^2^		BMI >27.57 kg/m^2^		*p*
		Mean	SD	Mean	SD	
Copeptin [pmol/l]	0′	7.9	8.7	5.0	4.0	0.418
	60′	6.7	6.5	4.3	3.0	0.275
	90′	6.6	6.4	3.8	2.5	0.110
	120′	10.1	10.1	4.0	4.0	**0.022**
	150	15.5	20.4	5.2	5.2	**0.018**
	180′	15.9	16.0	6.2	5.2	**0.036**
GH [ng/ml]	0′	1.8	2.0	2.0	7.1	**0.049**
	60′	2.3	4.2	2.9	6.1	**0.048**
	90′	3.3	3.1	2.7	5.6	0.054
	120′	9.5	7.0	3.5	5.6	**0.003**
	150′	12.4	9.5	3.1	4.7	**0.002**
	180′	7.0	5.1	3.5	3.6	0.050
ACTH [pg/ml]	0′	21.7	10.2	34.7	23.0	0.145
	60′	17.2	15.0	17.2	8.5	0.345
	90′	14.7	8.4	15.8	9.4	0.710
	120′	16.7	8.7	19.5	13.1	0.577
	150′	27.1	16.8	24.7	16.6	0.676
	180,	29.6	21.8	29.5	30.7	0.722
Glucose [mg/dl]	0′	78.6	8.7	82.7	7.4	0.132
	60′	111.0	40.0	137.6	45.8	0.058
	90′	85.1	29.7	116.8	39.2	**0.003**
	120′	67.6	9.5	92.2	27.2	**0.001**
	150′	62.6	9.6	77.0	19.1	**0.004**
	180′	67.8	11.0	70.5	12.9	0.844
Copeptin (area under the curve)		1536.0	1965.0	978.3	1034.7	**0.046**
ACTH (area under the curve)		4152.7	2499.7	5031.1	3132.6	0.496
GH (area under the curve)		924.6	726.0	361.8	395.5	**0.006**
Glucose (area under the curve)		12713.1	1257.2	14179.1	2303.3	**0.054**

Statistical significance values are highlighted in bold for better visibility

## Discussion

Recent findings on the stimulation of copeptin following an insulin tolerance test (ITT) [4, 17] suggest that copeptin may have a role not only in posterior but also anterior pituitary function. Using GST which is well accepted to assess GH but potentially as well adrenotropic function, we show here first data that copeptin is significantly stimulated in response to glucagon in controls and patients with intact posterior pituitary function. It appears to be linked to ACTH and in part to GH release following a GST which has been used to assess somatotropic and adrenotropic pituitary function [11, 13–17]. We show a parallel stimulation of copeptin and ACTH and only in subjects with normal posterior function a relation as well to GH release. Moreover, we demonstrated first evidence that the glucagon associated copeptin stimulation is modulated by BMI.

Currently, the mechanism through which glucagon stimulates ACTH and GH is unclear. A role of copeptin/AVP was proposed but has never been unequivocally established. It is known that AVP binds to three different receptors, V1a, V1b, and V2, where V1b receptor is expressed in the pituitary gland and pancreas, while AVP stimulates release of ACTH via V1b receptor. Interestingly, also insulin and glucagon secretions are mediated through V1b receptor [8, 18]. Furthermore, in stressful situations, such as in severe illness, the relationship between plasma osmolality and AVP is lost. This may rest on a synergistic action of CRH and AVP which stimulates ACTH and cortisol secretion [19, 20]. Under these circumstances, a correlation between copeptin (released in equimolar concentrations with AVP) and ACTH is plausible. Indeed, we have demonstrated a positive correlation between ACTH and copeptin at the time of maximal copeptin and ACTH release during GST in healthy controls. Though there are data that copeptin concentrations correlate with individual stress levels [21], the precise mechanism by which glucagon stimulates AVP/copeptin release is unclear. It may be directly stimulated or its release may be mediated though CRH or other mechanisms. Currently convincing evidence is lacking on the ACTH-releasing effects of intramuscular glucagon. Our results are well compatible with a direct effect. They contrast to a single study where glucagon and AVP apparently act additively on ACTH release arguing against an ACTH stimulation cascade via glucagon on AVP/copeptin to stimulate ACTH [22].

Our data clearly show that copeptin response following glucagon stimulation rests on an intact posterior pituitary function and is lost in patients with partial or overt diabetes insipidus. When combining all subjects with normal posterior pituitary function into one group, our data further suggest a weak correlation to GH release. This effect is clearly influenced by body weight as subjects with higher BMI show an attenuated copeptin response. This finding deserves further, more detailed studies as the mechanism of the attenuated GH response in obesity is not fully elucidated as yet [23, 24]. However, recent data confirmed a similar, lower GH response to GST in overweight/obese adult subjects with intact pituitary functions [25]. Thus, it appears plausible that glucagon acting via the release of AVP/copeptin from targets in the hypothalamus and posterior pituitary is involved in this regulation and copeptin response is attenuated in obesity. With higher than normal glucose levels during the first phase of the GST but significantly lower glucose concentrations in patients tested during an ITT, the mechanism is unlikely triggered by glucose [17, 23]. Unfortunately other factors known to be altered in obesity like insulin itself or FFA have not been measured as our study was not designed for this unforeseen interaction of copeptin with BMI. Glucagon, rapidly released in response to an insulin-induced hypoglycemia, may serve as a common link to unify results obtained with ITT and GST as previous data suggest that glucagon is rapidly released following ITT. This response critically depends on an intact hypothalamic function, particularly in terms of preserved CRH release [26], but this may not be the sole explanation as generation of hexarelin, a peptidyl fragment associated with the GH and ACTH-releasing activity or an induction of norepinephrine secretion in stimulating GH and ACTH release via α-receptors have been discussed [27, 28].

Finally, stress-related changes may bias our results. Indeed, severe stress as in myocardial infarction has been shown to acutely induce copeptin levels [29, 30]. Such a relationship is not confirmed in other stress conditions as under pathological gambling [31] or in tumor patients. The latter group frequently suffers of nausea, a symptom experienced as well as a short-term side effect of the GST [32]. However, when we systematically assessed a group of cancer patients concerning nausea and pain, we could not demonstrate any relation between the severity and copeptin concentrations [33]. Thus, even though we cannot rule out any stress or nausea induced influence on copeptin levels, such an influence is very unlikely to bias our results.

In summary, we have demonstrated a definite and unequivocal rise of copeptin concentrations during GST in subjects with satisfactory cortisol and GH responses, while copeptin responses were much lower and an increase was non-significant in patients with anterior pituitary dysfunction. Copeptin release depends critically on an intact posterior pituitary function, i.e., on preserved vasopressin/copeptin release from the posterior pituitary. Its release is attenuated in subjects with high BMI. The close correlation between copeptin and ACTH, but also the weaker association to GH, may indicate a stimulatory role and suggest a potential underlying mechanism for the release of anterior pituitary hormones in response to glucagon stimulation. We acknowledge, however, that these are preliminary data, and that the group of patients with pituitary disease was heterogenous. Hence, further studies might be useful, in particular in patients with pituitary disease who could be subdivided into groups with similar etiology (e.g., subject with non-secreting adenomas, subjects with acromegaly, prolactinomas, Cushing’s syndrome, etc.). It also remains to be established whether measurements of copeptin during evaluation of anterior pituitary function may add further information to clinical management of patients with pituitary insufficiency.

## Electronic supplementary material

Below is the link to the electronic supplementary material.

Supplementary material 1 (DOCX 16 kb)
