# Supplementary material for: Copeptin under glucagon stimulation
Source: Endocrine. 2015 Nov 17;52:344–51. doi: 10.1007/s12020-015-0783-7 (PMC4824796; doi:10.1007/s12020-015-0783-7)
Supplement: Supplementary file 1 — Supplementary material 1 (DOCX 16 kb) [file 12020_2015_783_MOESM1_ESM.docx]

**Supplementary Table A**. Comparison of hormonal parameters between Group 1, Group 2 and Group 3, where Group 1 - healthy controls – no pituitary disease, (n= 32), Group 2 - pituitary disease, but passed Glucagon Stimulation Test – GST (n=29) and Group 3 - pituitary disease failed GST – inadequate response of GH, cortisol or both (n=18).

| **Parameter** | **Group 1 (n=32)** | | | **Group 2 (n=29)** | | | **Group 3 (n=18)#** | | | **p** |
| --- | --- | --- | --- | --- | --- | --- | --- | --- | --- | --- |
|  | **Mean** | **SD** | **SEM** | **Mean** | **SD** | **SEM** | **Mean** | **SD** | **SEM** |  |
| **FSH [IU/L]** | 18.94 | 34.05 | 6.11 | 10.55 | 21.40 | 4.04 | 4.38 | 7.37 | 2.04 | 0.208 |
| **LH [IU/L]** | 13.70 | 16.54 | 3.02 | 6.09 | 8.65 | 1.63 | 1.92 | 2.75 | 0.73 | 0.007* |
| **Oestradiol [pg/mL]** | 73.47 | 91.16 | 18.23 | 74.35 | 62.86 | 16.80 | 40.25 | 41.88 | 14.80 | 0.540 |
| **Testosterone –female [ng/mL]** | 0.76 | 1.81 | 0.44 | 0.48 | 0.79 | 0.31 | 1.67 | 3.22 | 1.61 | 0.593 |
| **Testosterone –male [ng/mL]** | 2.99 | 2.55 | 1.04 | 4.14 | 2.89 | 0.87 | 1.46 | 2.02 | 1.43 | 0.910 |
| **Prolactin [ng/mL]** | 15.92 | 16.65 | 3.20 | 17.28 | 24.59 | 5.02 | 19.77 | 16.60 | 4.60 | 0.501 |
| **Free T3 [pg/mL]** | 4.05 | 4.42 | 0.85 | 3.10 | 0.62 | 0.12 | 2.59 | 0.51 | 0.12 | 0.219 |
| **Free T4 [ng/dL]** | 1.46 | 1.19 | 0.21 | 1.14 | 0.32 | 0.06 | 1.08 | 0.25 | 0.059 | 0.182 |
| **TSH [mIU/L]** | 1.78 | 1.35 | 0.24 | 1.09 | 0.85 | 0.16 | 1.16 | 1.29 | 0.33 | 0.062 |

*Statistically significant difference between Group 1 and Group 2 versus Group 3. # Most patients in Group 3 received some form or replacement therapy, i.e.), L-thyroxine (n=12), intramuscular testosterone (n=2) or oestrogens±progestin (n=3).

**Reference ranges**: TSH 0.27-4.2 mIU/l, free T4 0.93-1.7 ng/dl, free T3 2.6-4.4 pg/ml, Testosterone (men) 2.8 – 8.0 ng/ml, testosterone (women) 0.084-0.481 ng/ml, prolactin 3.9-25.4 ng/ml.

**Supplementary Table B**

Spearman correlation coefficients between maximal increases of copeptin concentrations [copeptin delta (Δ)] and maximal increases of ACTH or cortisol concentrations (ACTH Δ and cortisol Δ). Group 1 (healthy controls, n=32), in Group 2 (history of pituitary disease, but passed GST, n=29), Group 3 (unsatisfactory response during GST for GH, cortisol, or both, n=18).

| **Group** | **r_copeptinΔ-ACTHΔ_** | **p_copeptinΔ-ACTHΔ_** | **r_copeptinΔ-cortisolΔ_** | **p_copeptinΔ-cortisolΔ_** | **r_copeptinΔ-GHΔ_** | **p_copeptinΔ-GHΔ_** |
| --- | --- | --- | --- | --- | --- | --- |
| **1** | 0.653 | ***0.0001*** | 0.673 | ***<0.0001*** | 0.284 | 0.1281 |
| **2** | 0.170 | 0.3956 | 0.0473 | 0.8075 | 0.174 | 0.3670 |
| **3** | -0.241 | 0.3516 | -0.0279 | 0.9124 | -0.0951 | 0.7261 |
